# Data on tree height and diameter for *Pinus kesiya* in Zambia

**DOI:** 10.1016/j.dib.2019.104199

**Published:** 2019-08-05

**Authors:** Phillimon Ng'andwe, Donald Chungu, Arthur M. Yambayamba, Alice Chilambwe

**Affiliations:** a Copperbelt University, School of Natural Resources, P.O. Box 21692, Kitwe, Zambia; b Copperbelt University, Directorate of Distance Education and Open Learning, P.O. Box 21692, Kitwe, Zambia

**Keywords:** Rotation, Pine trees, *H-d* model, Data

## Abstract

Forest inventories in plantations of non-native trees are conducted every five years in Zambia. Characteristics of data collected through these inventories are presented here. The data includes diameter at breast height (*d*), total tree height (*h)* and rotation categories for trees sampled. This data supported the development of robust *h-d* models for planted *Pinus kesiya* in the country. We have also presented graphical visualization of the composition and trends of the data by site and rotation. Datasets were filtered and cleaned and are ready to be used for other purposes in order to improve understanding of *P. kesiya* growth. For more insight please see “Modeling the height-diameter relationship of planted *Pinus kesiya* in Zambia” (Ng’andwe et al., 2019).

**Table:** Specifications Table

Subject area	Forestry growth modeling
More specific subject area	Height-diameter model for tropical non-native *Pinus kesiya*
Type of data	Table, graph, figure
How data was acquired	Data was collected during forest plantations inventories in Copperbelt province in Zambia. We sampled 7,691 trees from temporal random sample plots for model development and 5,301 trees for model validation. Data collection for model development and model validation was conducted at different measurement occasions five years apart.
Data format	*Raw, filtered, analyzed*
Experimental factors	Data presented constitute pairs of diameters and heights of trees. For development data, we present data as: (i) first and second rotation categories, (ii) site specific data and (iii) combined data (i.e. data irrespective of site and rotation categories). Height-diameter model development was based on the combined data of *P. kesiya*. The validation data presented does not include additional categories apart from *d*.
Experimental features	We selected eight popular theoretical functions used in forest growth modeling selected from literature on the basis of simplicity, biological logic and reliability. These models were fitted to the datasets in order to choose the most appropriate function for the development of robust *h-d* models for *P. kesiya* in Zambia. The statistical performance measures and goodness-of-fit for the models were computed along with model diagnostics
Data source location	Copperbelt province, Zambia
Data accessibility	All data used and generated is included in this article
Related research article	P. Ng'andwe, D. Chungu, A.M. Yambayamba, A. Chilambwe, Modeling the height-diameter relationship of planted *Pinus kesiya* in Zambia. *Forest Ecology and Management*, 447 (2019) 1–11.

**Table:** 

**Value of the data** • This is data will enhance the development and comparisons of tropical pine height-diameter models for prediction in the region and globally. • The composition of data presented include the first and second rotation of *P. kesiya* suitable for tree growth modeling of successive plantations. • This data also creates an opportunity to improve further the developed h-d model for *P. kesiya*. The approach used is simplified based on diameter as the predictor variable, hence Forest Managers will find this data and developed models potentially user friendly. • The data can be used for generating height-diameter curves for different rotations, site quality assessments and for developing biomass equations for *P. kesiya*

## 1 Data

**Fig. 1:**
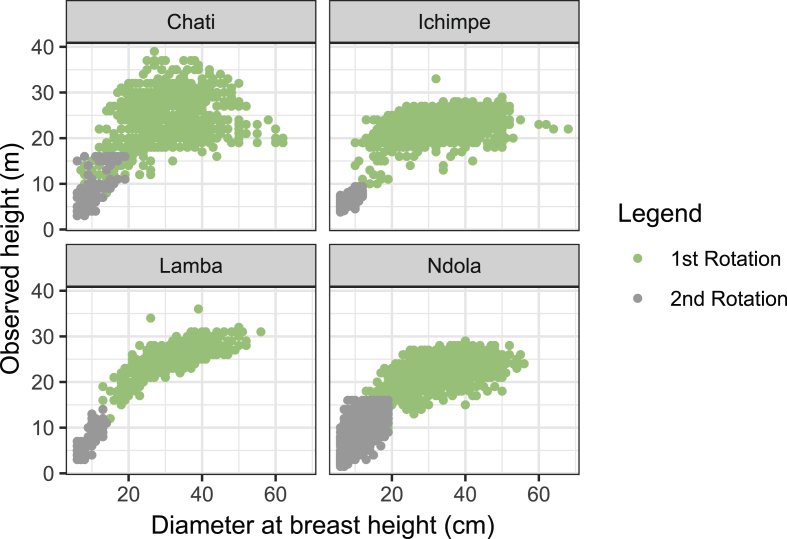
Development data composition in Chati, Ichimpe, Lamba and Ndola sites for planted *Pinus kesiya.* The green and grey dots are for the first and second rotations, respectively.

**Fig. 2:**
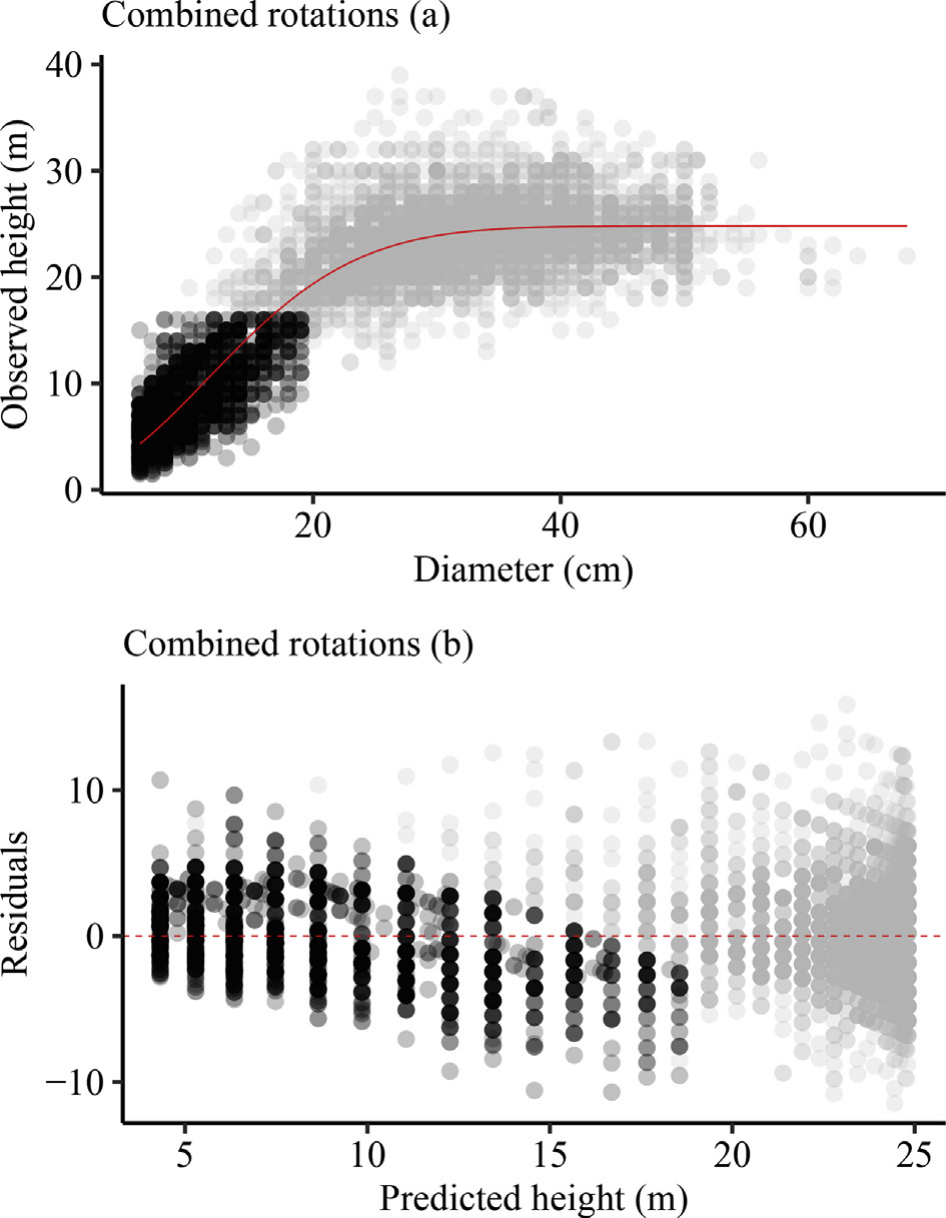
Fitting of the country-wide model on the combined data of *Pinus kesiya* in Zambia: (a) Scatter plots of observed height against diameter at breast height overlaid with the curve (solid red line) produced by the country *h-d* model (equation (1)) and (b) Plot of residuals against predicted height. Grey dots refers to first rotation and black dots to the second rotation.

**Fig. 3:**
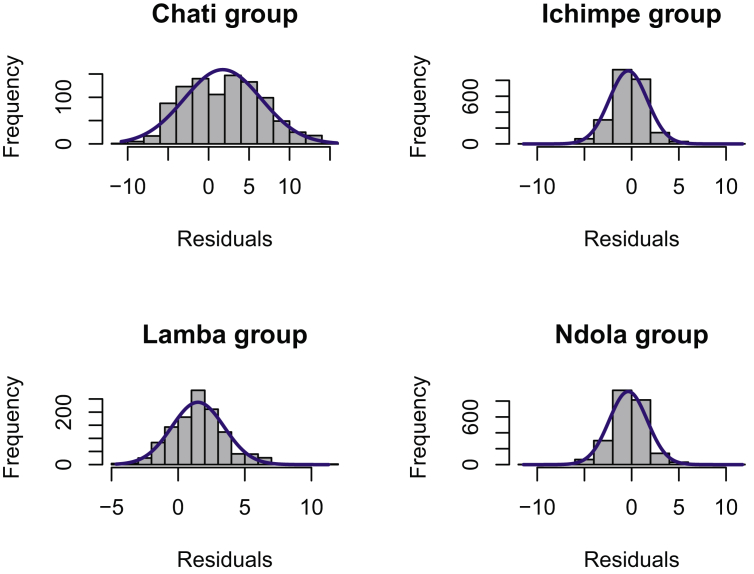
Test of normality of residuals for country-wide model fitted to the *Pinus kesiya* site specific data. The bars are the observed compared with blue smooth line representing a normal distribution.

**Fig. 4:**
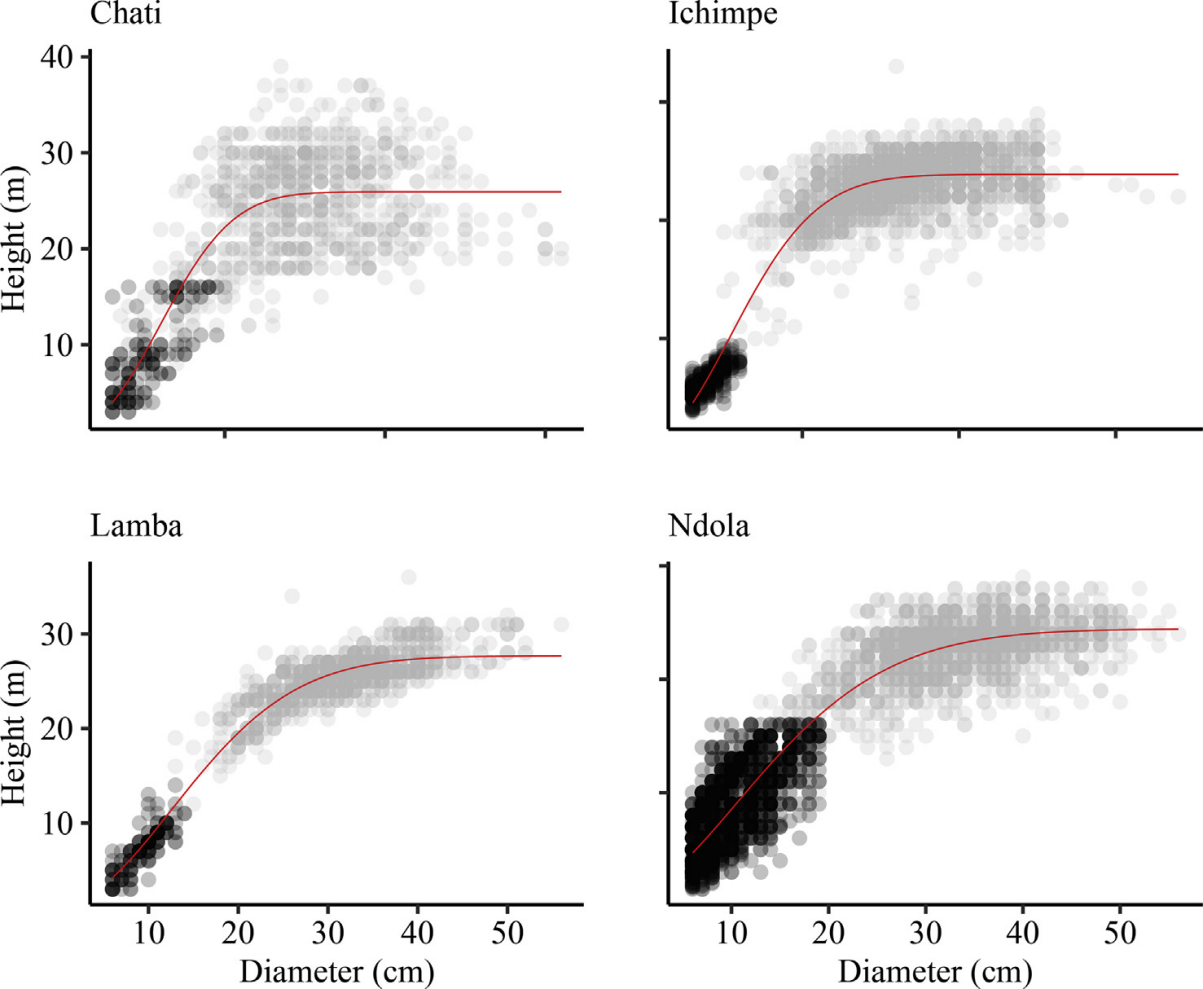
Fitting of the country-wide model on the site specific data of *Pinus kesiya* in Zambia. The model seem to have fitted well on the graph for each site but the highest accuracy of statistic fits was observed in Ichimpe site (MAPE = 8.7%) and lowest in Ndola (MAPE = 21.7%) as shown in Table 4. Grey and black shades represent first and second rotation data, respectively.

**Fig. 5:**
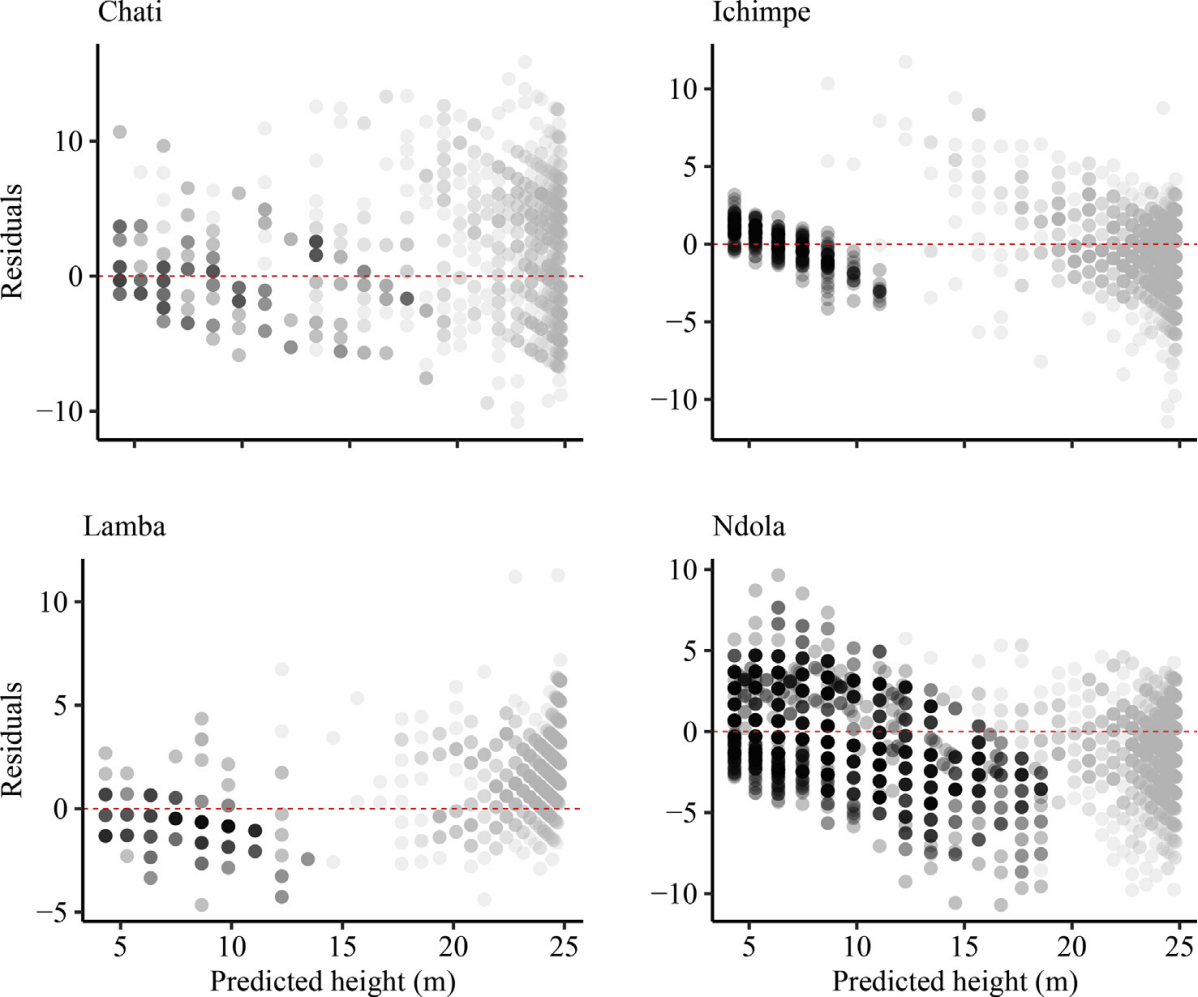
Plot of residuals vs predicted height of *Pinus kesiya h-d* model in Chati, Ichimpe, Lamba and Ndola sites. Grey and black dots represent the first and second rotation, respectively.

*Pinus kesiya* is one the fast growing non-native trees of economic importance in Zambia and the region. Height-diameter modeling is important in the prediction of forest growth, biomass and carbon. Data presented was collected from inventories in the Copperbelt province in Zambia. These data include: number of trees sampled, mean tree height and mean diameter at breast height in four different plantation sites. The data were categorized as (i) first rotation, (ii) second rotation and (iii) combined (Table 1). First rotation refers to the first *P. kesiya* trees that were planted after removing the native vegetation and are usually above 25 years old. Second rotation refers to the *P. kesiya* trees that were planted immediately after the first rotation trees were harvested and are less than 25 years of age. Data on rotation is related to age obtained from administrative records i.e. the year when trees were planted in the field to the year when the inventory was conducted. We used 7,691 trees with complete *h* and *d* pairs in model development [1]. The data composition in each group is presented in Fig. 1. The combined data was used to develop the model parameter estimates (Table 3). The model fit to the combined dataset and *h-d* curve produced by the country level model (Equation (1)) and associated plots of residuals against predicted height are presented in (Fig. 2) and normality checks (Fig. 3). We also fitted the country-level model to site data and generated site-specific *h-d* models (Fig. 4) and homoscedasticity diagnostics checks (Fig. 5). Parameter estimates for site specific models and fit statistics are presented in Table 4. Data presented at site level includes plots of residuals versus predicted height to check for normality and homoscedasticity of errors that could influence parameter estimates and fit statistics. A megaphone pattern would reveal heteroscedasticity which is more related to the response variable *h* [2]. Data related to the comparison of the country level model and site-specific model on the basis of the mean relative error (MRE) and mean absolute percent error (MAPE) is also presented in Table 4.

**Table 1:** Characteristics of data used in this study for *Pinus kesiya* in Zambia. Numbers in brackets represent standard deviation of the mean. Data, irrespective of first and second rotation is indicated as ‘combined' and was used in model development. First rotation refers to characteristics of data collected from trees above 25 years old and second rotation from trees below 25 years old. Data used for validation of models was only available as “combined” irrespective of site and rotation.

Site	Combined				First rotation			Second rotation		
	No. plots	a*N*	Mean *d*, cm	Mean *h,* m	*N*	Mean *d,* cm	Mean *h*, m	*N*	Mean *d,* cm	Mean *h*, m
Chati	527	952	27.2 (11.1)	22.3 (7.6)	826	29.8 (9.5)	24.4 (5.6)	126	10.4 (3.5)	8.7 (4.1)
Ichimpe	1738	2321	28.5 (11.9)	20.3 (6.5)	1940	32.7 (8.5)	22.9 (2.4)	381	8.0 (1.6)	6.5 (1.1)
Lamba	544	1169	28.8 (7.80)	23.1 (6.5)	1027	31.5 (6.9)	25.3 (2.8)	142	9.4 (2.1)	7.2 (2.4)
Ndola	1359	3249	21.5 (12.9)	15.7 (7.7)	1621	33.0 (7.8)	22.5 (2.8)	1,628	10.1 (3.3)	8.4 (3.5)
**Fit-data**	**4168**	**7691**	**25.6 (12.4)**	**18.9 (7.8**)	**5414**	**32.1 (8.2)**	**23.5 (3.4**)	**2,277**	**9.7 (3.1**)	**8.1 (3.3**)
**V/data**^b^	**3211**	**5301**	**32.3 (8.4)**	**23.8 (3.4)**	**-**	**-**	**-**	**-**	**-**	**-**

a *N* is number of trees, *d* is diameter at breast height, and *h* is tree height

b V/data refers to independent validation data

**Table 2:** Theoritical functions used in the development of *h-d* models for *Pinus kesiya* in Zambia.

No.	Function name	*K*^a^	Function^a^	References
1	Näslund	2	$h=1.3+\frac{d^{2}}{{\left(\beta_{1}+\beta_{2}d\right)}^{2}}$	Pukkala et al., (1990)
2	Power	2	$h=1.3+\beta_{1}d^{\beta_{2}}$	Eerikäinen (2003)
3	Curtis	2	$h=1.3+\beta_{1}{\left(\frac{d}{1+d}\right)}^{\beta_{2}}$	Saramaki (1992)
4	Chapman-Richards	3	$h=1.3+\beta_{1}{(1-\text{exp}\left(-\beta_{2}d\right))}^{\beta_{3}}$	Lumbres et al. (2013)
5	Weibull	3	$h\;=1.3+\beta_{1}(1-\text{exp}\left(-\beta_{2}d^{\beta_{3}}\right))$	Huang et al. (1992)
6	Modified Logistic	3	$h=1.3+\frac{\beta_{1}}{1+{\beta_{2}}^{-1}d^{-\beta_{3}}}$	Lumbres et al. (2013)
7	Exponential	3	$h=1.3+\beta_{1}\times {\text{exp}}^{\frac{\beta_{2}}{\left(d+\beta_{3}\right)}}$	Huang et al. (1992)
8	Hossfeld	3	$h=1.3+\frac{d^{\beta_{1}}}{\beta_{2}+\beta_{3}d^{\beta_{1}}}$	Sharma (2009)

a *k is the* number of fixed model parameters, h is the total tree height in m, *d is* the diameter at breast height in cm, and the fixed model parameters are.$\beta_{1},\;\beta_{2}\text{and}\;\beta_{3}$.

**Table 3:** Estimated parameters and their associated statistic fits for each *h-d* model for *Pinus kesiya* in Zambia.

Model based on	Parameter estimates^a^			Statistic fits^a^							
				Modeling data				Validation data			
	$\beta_{1}$	$\beta_{2}$	$\beta_{3}$	MAPE	RMSE	MPA	Rank	MAPE	RMSE	MPA	Rank
Näslund	0.1581 (0.006)	1.7503 (0.0164)		20.12	3.31	10.95	7	9.78	2.92	8.51	7
Power	2.5490 (0.041)	20.116 (0.0058)		25.56	3.86	14.88	8	11.81	3.46	11.99	8
Curtis	0.7566 (0.156)	3.0575 (0.1177)		17.71	3.16	9.96	6	9.19	2.76	7.61	6
Chapman-Richards	24.217 (0.084)	0.1257 (0.0022)	3.4682 (0.093)	15.94	2.96	8.74	2	8.02	2.49	6.19	2
Weibull	23.520 (0.065)	0.0041 (0.0002)	1.9650 (0.021)	15.63	2.92	8.51	1	7.96	2.48	6.15	1
Modified Logistic	25.280 (0.124)	0.0011 (0.0001)	2.5950 (0.036)	15.99	2.98	8.85	4	8.04	2.49	6.20	3
Exponential	32.007 (0.024)	−10.761 (0.2597)	−1.9775 (0.144)	17.18	3.10	9.65	5	8.69	2.63	6.92	5
Hossfeld	2.5950 (0.036)	36.910 (3.2720)	0.0396 (0.002)	15.90	2.98	8.90	3	8.19	2.55	6.52	4

a Estimated parameters for each developed model are indicated by $\beta_{1},\;\beta_{2}\text{and}\;\beta_{3}$, standard errors are in parentheses, MAPE is mean absolute percent error, RMSE is root mean square error, MPA is model prediction accuracy. Note that a model based on Weibull theoretical function ranks number 1 during modelling and validation based on the statistic fits.

**Table 4:** Parameter estimates and their statistic fits for *h-d* models specific to site for *Pinus kesiya* in Zambia.

Site	Model	Parameter estimates and their standard errors^a^			Statistic fits^b^	
		$\beta_{1}$	$\beta_{2}$	$\beta_{3}$	MRE	MAPE
Chati	Country	23.52 (0.065)	0.0041 (0.0002)	1.965 (0.021)	−0.03	19.9
	Site	24.62 (0.229)	0.0018 (0.0006)	2.320 (0.128)	0.05	20.0
Ichimpe	Country	23.52 (0.065)	0.0041 (0.0002)	1.965 (0.021)	0.02	8.7
	Site	22.57 (0.068)	0.0043 (0.0030)	1.990 (0.287)	0.01	8.5
Lamba	Country	23.52 (0.065)	0.0041 (0.0002)	1.965 (0.021)	−0.04	9.5
	Site	26.37 (0.125)	0.0040 (0.0004)	1.918 (0.033)	0.11	13.4
Ndola	Country	23.52 (0.065)	0.0041 (0.0002)	1.965 (0.021)	0.10	21.7
	Site	23.14 (0.137)	0.0070 (0.0010)	1.687 (0.024)	0.06	20.4

a Estimated parameters for *h-d* models specific to sit and rotation are indicated by$\;\beta_{1},\;\beta_{2}\text{and}\;\beta_{3}$, Overestimation and underestimation are indicated by positive and negative values respectively.

b MRE is the mean relative error, and MAPE is the mean absolute percent error.

## 2 Experimental design, materials, and methods

### 2.1 Data acquisition

Data presented was collected during the forest plantation inventory in 2011 and 2016. All compartments were assessed. The equipment used included diameter tapes (for measuring *d*) and Sunnto clinometers (for measuring *h*). Data presented was filtered from the main inventory database and prepared for modeling. We present 7,691 trees of *P. kesiya* for model development and 5,301 trees for validation.

### 2.2 Data exploration

The collected raw data was subjected to cleaning and generating of preliminary descriptive statistics in Microsoft Excel and saved in csv (Comma delimited) format. We used R to develop basic graphical and numerical diagnostics [3]. We checked for the normality of data to confirm if the assumptions for parametric tests were met by using both graphical and numerical measures (Fig. 3).

### 2.3 Height-diameter model development

We selected from literature eight model functions popular in forestry modeling (i.e. Näslund, Power, Curtis, Chapman-Richards, Weibull, Modified Logistic, Exponential and Hossfeld) for model development [4], [5], [6], [7], [8] (Table 2). These functions were fitted to *P. kesiya* data using nls function in R. Actual datasets used are stored in a separate raw data file (pkesiya_fitdata.csv and pkesiya_validationdata.csv). We followed established procedures during fitting, parameterization and validation [5], [9], [10]. For more information please see “Modeling the height-diameter relationship of planted *Pinus kesiya* in Zambia” [1].

### 2.4 Performance analysis

All models were subjected to statistical and graphical performance tests [11], [12]. Consistent with recommended practices in forestry modeling [2], [9], we also conducted model diagnostic checks such as testing for normality and homoscedasticity of residuals for different models fitted to the data. The graphical performance of the best model when fitted to the combined data is shown in (Fig. 2a) and plot of residuals against predicted height in Fig. 2b. Data was not split for model development and validation, instead an independent data was collected for validation purpose. The performance of developed models were evaluated numerically: Relative error (RE), mean relative error (MRE), absolute percent error (APE), mean absolute percent error (MAPE), Root mean square error (RMSE), model prediction accuracy (MPA) (Table 3) and graphically (Fig. 2a and b). The best model was based on its consistency and final ranking based on MAPE, RMSE and MPA goodness of fit criteria (Table 3). Parameter estimates for the models, performance evaluation and model ranks are presented in Table 3. On the basis of model ranking, the best *h-d* model for *P. kesiya* based on the Weibull function (Table 2, No. 5) is presented as the country model (equation (1))

(1) $$h\;=1.3+23.5200*(1-\text{exp}\left(-0.0041*d^{1.965}\right))$$

Further model tests on the country-level model (equation (1)) were performed to evaluate the influence of site and/or rotation on the prediction accuracy using ANOVA. Prior to conducting ANOVA, residuals were subjected to normality and homogeneity of variance tests. In this regard, we performed Shapiro-Wilk test and also visualized the distribution of residuals using histograms were necessary. Residuals with a normal distribution would be indicated by a higher value (W) of Shapiro (W > 0.05) and a higher value of (*p*) (Shapiro *p* > 0.05). However, any model with a high number of observations may yield a significant *p*-value (*p* < 0.05) for the Shapiro–Wilks test [12]. Therefore, we also used visual inspection of the histogram and if skewed, data was transformed to comply with the assumptions of ANOVA.

In some cases where variances were not homogenous after performing the Bartlett homogeneity test, a Welch t-test for unequal variance was used instead [12]**.** Multiple pairwise comparisons among the levels of site was conducted using least-squares means (*lsms*) procedures for all significant effects on RE and MAPE for datasets with unequal variance [12], [13]. Depending on the outcome of the analysis, site specific or rotation specific *h-d* models were developed as submodels of equation (1) (Table 4). We again used the mean relative error (MRE) and MAPE to evaluate the performance of site-specific modes. Models that passed this final step were considered for *h* estimation at the site and/or rotation level for *P.kesiya* in Zambia [1]. Equations used in the evaluation process are detailed in Table 5. The R packages that we utilized included *Metrics* for statistical performance tests, *ggplot2* and *gridExtra* for graphics, *dplyr* for sub sampling of data, among others [3].

**Table 5:** Formulae used in the numerical and graphical height-diameter model evaluation for *Pinus kesiya* - Zambia.

No.	Name of metric	Abbreviation	Formulae	Purpose
1a	Relative error	RE	RE_*i*_ = $\;\frac{{\hat{h}}_{i}-h_{i}}{h_{i}}$	Precision
1b	Mean relative error	MRE	MRE = $\frac{1}{n}\sum_{i=1}^{n}\frac{\left({\hat{h}}_{i}-h_{i}\right)}{h_{i}}$	Precision
2a	Absolute percent error	APE	${\text{APE}}_{i}=\frac{\left|h_{i}-{\hat{h}}_{i}\right|}{h_{i}}\times 100$	Accuracy
2b	Mean absolute percent error	MAPE	MAPE = $\frac{100}{n}\sum_{i=1}^{n}\frac{\left|\left(h_{i}-{\hat{h}}_{i}\right)\right|}{h_{i}}$	Accuracy
4	Mean prediction bias	MPB	$\text{MPB}=\frac{1}{n}\sum_{i=1}^{n}\left(h_{i}-\hat{h_{i}}\right)$ = $\sum_{i=1}^{n}\frac{\left(h_{i}-{\hat{h}}_{i}\right)}{n}$	Reliability
5	Root mean square error	RSME	RMSE $=\sqrt{1/\left(n-k\right)\overset{n}{\underset{i=1}{\sum }}{\left(h_{i}-{\hat{h}}_{i}\right)}^{2}}$	Accuracy
6	Model Prediction accuracy	MPA	${\text{MPA}=\text{MPB}}^{2}+{\text{SD}}^{2}$	Reliability

Where, RE_*i*_ is the relative error and MRE is the mean relative error obtained by diving RE by the total number of measured trees, *n*. APE_*i*_ is the absolute percent error, MAPE is the mean absolute percent error (i.e. an everage of APE_*i*_), $h_{i}$ is the measured tree height for the ith tree; ${\hat{h}}_{i}$ is the predicted tree height for the ith tree; MPB is the mean prediction bias (i.e. the error associated with prediction for the ith tree which reflects the deviation of the model with respect to the measured value); SD is the standard deviation of the prediction bias; RMSE is the root mean square error; MPA is the model prediction accuracy which combines mean prediction bias an the standard deviation of residuals; k is the number of fixed model parameters.
